# Ethical framework for FACILITATE: a foundation for the return of clinical trial data to participants

**DOI:** 10.3389/fmed.2024.1408600

**Published:** 2024-07-17

**Authors:** Ciara Staunton, Johanna M. C. Blom, Deborah Mascalzoni, on behalf of the IMI FACILITATE Consortium

**Affiliations:** ^1^Eurac Research, Institute for Biomedicine, Bolzano, Italy; ^2^School of Law, University of Kwazulu-Natal, Durban, South Africa; ^3^Department of Biomedical, Metabolic and Neural Sciences, University of Modena and Reggio Emilia, Modena, Italy; ^4^Center for Neuroscience and Neurotechnology, University of Modena and Reggio Emilia, Modena, Italy; ^5^Center for Research Ethics and Bioethics, Department of Public Health and Caring Sciences, Uppsala University, Uppsala, Sweden

**Keywords:** health data-sharing, clinical trials, decision-making, informed consent, return of clinical trial data

## Abstract

This paper discusses the importance of return of clinical trial data to patients in the context of the FACILITATE project that aims to develop a participant-centric approach for the systematic return of individual clinical trial data. It reflects on the need for an ethical framework to support the return of clinical trial data. The discussion revolves around the developing FACILITATE ethical framework, specifically focusing on the ethical principles that form the foundation of the framework and guidance on how to implement those principles into practice.

## Introduction

Substantial volumes of health data are generated throughout a clinical trial that can prove valuable to the study participants and equally, data relevant to study participants may emerge post-trial. Sharing individual data with study participants can prevent redundant, potentially invasive, and costly medical examinations, benefiting both the participants and society. The individual data generated during the clinical trial can contribute to more informed healthcare decisions. Therefore, timely communication of these data to study participants can have a significant impact on their health (1).

Returning individual clinical trial data to study participants upholds their role in clinical trials and their autonomy. It also empowers them to make informed healthcare choices. However, patients report that the routine return of individual clinical trial data to study participants is currently uncommon, especially after completion of the trial (2, 3). This is partly due to the fact that it is unclear who holds responsibility for this return: pharmaceutical companies are not allowed to directly contact study participants post-trial, hospitals may not have resources to handle long term return of data, and there are no acknowledged common pathways for technological solutions for secure data return or the disclosure of aggregated clinical trial results on public platforms as required by regulations (2).

Despite the current practical barriers, study participants have the right to access their personal data. They hold a legal right under the General Data Protection Regulation (GDPR), (4) and more broadly, they have a moral right to this information. Having this information can avoid unnecessary duplicate and invasive tests and demonstrates reciprocity by acknowledging the important role of patients. Indeed, initiatives such as Transcelerate^1^ (5) and the Multi-Regional Clinical Trials (MRCT) Center of Brigham and Women’s Hospital and Harvard^2^ (6) are initiatives that are moving toward making data available. However, the operationalization of this right is hampered due to the differing approaches at a national level regarding the requirements on the return of data, that can vary according to the data type (e.g., return of genetic data must be done by a genetic counselor in France).

Such initiatives are just the beginning, and it is crucial to establish processes to overcome the complexities in returning clinical trial data. The FACILITATE project aims to develop a participant centric approach for the systematic return of individual participant clinical trial data. It envisions a bottom-up, participant-centric process that empowers study participants to exercise more control over their healthcare decision-making while balancing other interests, including the scientific integrity of the trials (7). This approach is expected to assist both private and public researchers in navigating the ethical complexities associated with returning individual clinical trial data to participants.

To create a participant-centric ethical ecosystem centered around participants, FACILITATE must exceed these minimum standards. FACILITATE aims to actively engage participants as equal partners. This paradigm is rooted deeply in recent literature on patient involvement, engagement, and empowerment, which underscores the value of treating participants not as passive subjects but as active contributors to the research process (8–10).

Our definition of ‘participant-centric’ builds on contemporary discussions that emphasize the collaborative creation of a clinical trial ecosystem that integrates the perspectives of patient representatives collected throughout the development process, moving toward a model of shared decision-making where patients’ voices and choices are central (8, 11, 12). Examples from the latest literature include initiatives that focus on patient engagement through advisory panels where trial participants contribute to study design and implementation strategies. These frameworks not only enhance the relevance and acceptability of research but also improve patient outcomes by aligning the studies more closely with participant needs and expectations (13, 14).

The FACILITATE participant-centric approach means the co-creation of a clinical trial ecosystem with patients’ representatives and patients’ perspectives (collected throughout the development of the process). Participant centric to us means not only informed by participants but represents a cultural shift from the paternalistic paradigm of “we know what is best for patients” toward a “let us build the system together.”

Also, the participant-centric model proposed in FACILITATE is designed to ensure that all aspects of the trial—from the design, through to the execution, and the post-trial phase—respect and incorporate the insights and preferences of participants. This includes regular and transparent communication, personalized data return, ensuring that participants can access their data in a manner that is both meaningful and respectful of their autonomy (15, 16).

An integral part of FACILITATE is identifying a set of ethical principles and a framework for implementing these principles to guide the return of individual participant data and pseudonymized data re-use for future research. Such a framework sets out the expectations around clinical trial data use and provides much needed guidance to fill the gaps to current legal frameworks and guidance. To succeed, this ethical framework must be co-created with stakeholders participating in the clinical trial, in particular patients. It is for their benefit that clinical trial data should be returned and reused, thus the processes should better reflect the needs and expectations of patients. Such an approach in development can ensure that the ethical frameworks steer us toward processes that prioritize participant-centricity while acknowledging the diverse contexts in which clinical trials may occur. This participant-centric orientation serves as the foundation for the processes developed within FACILITATE, including the ethical frameworks, prioritizing participants’ agency over data decisions.

The ethical concerns and challenges that arise in the return of individual clinical trial data and the secondary use of pseudonymised clinical trial data are distinct. At an early stage it was decided to develop two distinct ethical frameworks for FACILITATE: an ethical framework on the return of individual participant clinical trial data and an ethical framework on the reuse of pseudonymised clinical trial data. It is the ethical framework on the return of individual clinical trial data that is the subject of this policy brief.

## Current policy options and implications

### Reflection on current policy options

The existing regulatory framework for clinical trials, particularly regarding informed consent and data sharing, is multifaceted and evolving. Regulations such as the General Data Protection Regulation (GDPR) and various national and international guidelines (e.g., the Declaration of Helsinki, Declaration of Taipei and CIOMS Guidelines) govern how data must be handled, emphasizing the protection of participant data and the necessity for explicit consent. However, these regulations often present challenges, particularly in the context of dynamic, data-intensive research environments where the needs for flexibility and participant engagement are increasingly recognized. Key issues include the static nature of traditional informed consent forms, the complexity of explaining data reuse, and the implications of emerging data technologies that may outpace current consent practices.

### FACILITATE’s methodology and contributions

**Figure 1:**
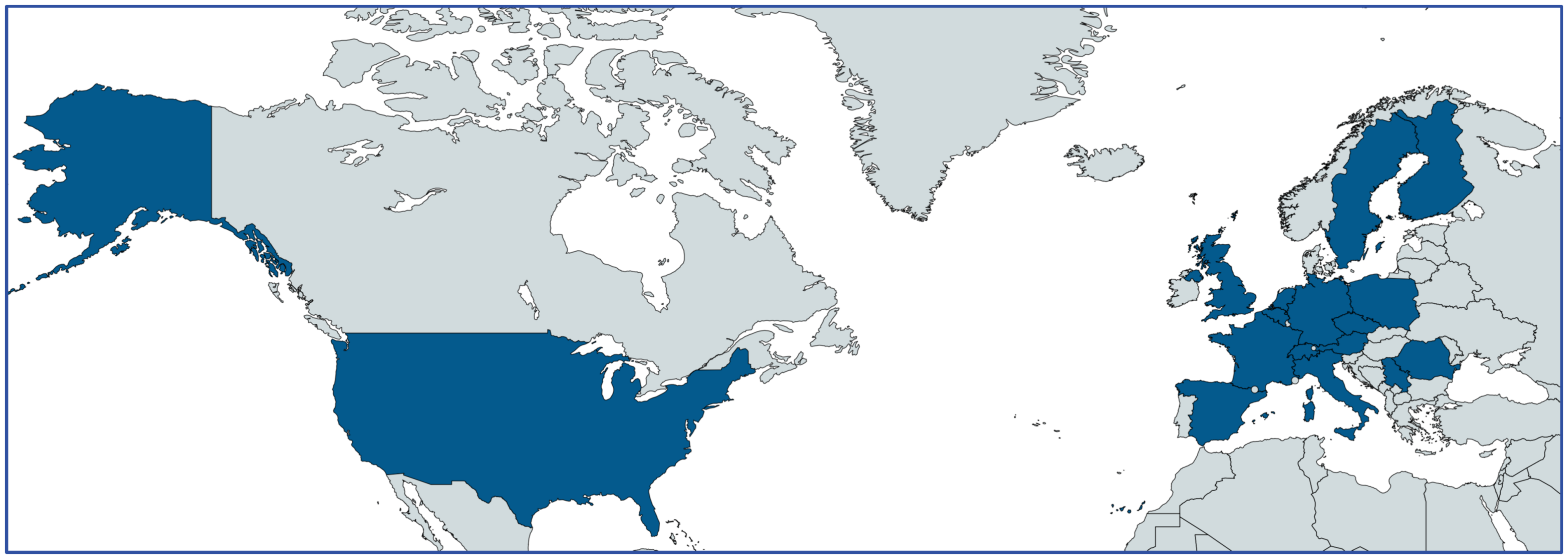
Composition of FACILITATE.

FACILITATE is not starting its work in a vacuum. It builds upon the foundation laid by preceding projects, ethical frameworks, regulations, and guidelines (as outlined in Table 1), empirical research, published conceptual analyses, industry initiatives toward patient-centered approaches, and efforts to establish procedures for returning individual clinical trial data to participants (such as the TransCelerate iPDR, jointly developed with patient groups). FACILITATE has adopted the methodology of reflective equilibrium (17). This approach follows the path of reflection, discussion with all stakeholders (Figure 1 for details) and revision to reach a contextualized conclusion that goes beyond pure academic analysis. Our stakeholders include patients, industry, academia, and we have deliberation on these issues through a series of consultative meetings with patients, as well as regular online and in person meetings as a consortium. This aligns with the participant centric approach of FACILITATE and reflects the cocreation approach that is central to the project.

**Table 1:** Instruments analysed for return of data.

CIOMS	International ethical guidelines for health-related research involving humans
MRCT	Return of individual results to participants recommendations document
American College of Medical Genetics and Genomics	Recommendations for reporting of secondary findings in clinical exome and genome sequencing
World Medical Association	Declaration of Helsinki
World Medical Association	Declaration of Taipei
UNESCO	Universal declaration on human rights and the human genome
UNESCO	International declaration on human genetic data
ICH	Guideline for genomic sampling and management of data
Council of Europe	Additional protocol to the convention on human rights and biomedicine, concerning biomedical research
Council of Europe	Recommendation (2006) 4 of the committee of ministers to member states on research on biological materials of human origin
Council of Europe	Oviedo convention
European Commission	General data protection regulation
European Commission	Clinical trials regulation
European Commission	Draft regulation for a European health data space
National Academy of Sciences	Returning individual-specific research results to participants: guidance for a new research paradigm
Global Alliance for Health	2021 Policy on clinically actionable genomic research results
OECD	Recommendation on health data governance

The FACILITATE project encompasses a diverse partnership network consisting of 29 partners from both within the EU—specifically from Italy, Austria, Belgium, Czech Republic, Finland, France, Germany, Poland, Romania, Spain, and Sweden—and outside the EU, including Serbia, Switzerland, the UK, and the USA.

Funding from the IMI2_JU supports 17 of these partners, including three universities, one research center, six hospitals, two representative patient groups [formed by rare disease and non-rare disease expert patients, and four small and medium-sized enterprises (SMEs)]. The remaining 12 partners, comprising 10 from the pharmaceutical industry under the European Federation of Pharmaceutical Industries and Associations (EFPIA) and two data analytics firms, do not receive direct funding.

Also, the dual approach of reflecting on current policies and actively shaping new methodologies through FACILITATE positions the project as a transformative force in clinical trial data management. This approach not only addresses existing policy gaps but also actively contributes to the evolution of ethical standards in clinical research, ensuring that the rights and needs of participants are at the center of data governance practices.

A starting point in the process was a review of current regulations, guidelines, frameworks, and participants and public’s views that impact directly or indirectly on building an ethical framework for the return and reuse of individual research data (Table 1). Following this, an initial list of key ethical principles was identified: respect for persons and community; beneficence; privacy; utility; empowerment; public interest; transparency; and accountability. These principles were selected as they were deemed to be principles as described in the documents that would best support FACILITATE’s participant centric approach.

The next step in the process was to understand how these documents and policies are perceived, understood and applied in practice. Informed consent stands as an enduring legal and ethical imperative that must be adhered to in research endeavors, including clinical trials.

It empowers research participants to exercise their autonomous choice regarding the utilization of their body and health data (18, 19) The current landscape of informed consent is in a state of ongoing evolution with many criticisms revolving around the lengthy and complicated nature of informed consent forms, but also that the consent is static (18). In the context of today’s data-driven world, where data can be readily reused and shared for diverse purposes, there are concerns that the traditional informed consent process may no longer be suitable (20–23).

A further consideration that is pertinent for FACILITATE is that legal constraints often hamper the effective informing of participants and render them too intricate to genuinely raise awareness among participants about their involvement in research (4). We have thus lost sight of half the purpose of the informed consent process, that is to inform participants.

FACILITATE is developing its processes within a changing regulatory landscape, keeping an eye on developments like the draft European Health Data Space (EHDS), which will affect the governance of clinical trial data use if it comes into force, but of which there are some ethical concerns in the proposed processes (24). Research shows that participants have varying preferences regarding the reuse and sharing of their data, depending on the research purpose and data user, and the fact that these preferences can evolve over time (25, 26). Similarly, individualized preferences exist regarding the return of results, and these preferences may change as well. Those preferences are well rooted into feelings of fear and mistrust that should not be overlooked as lack of trust is a growing issue that is leading to decreased participation in clinical trials (27).

There is a growing recognition that we may need to adjust the mechanisms governing data sharing in research. While the current informed consent processes employed by sponsors may not align with the needs of contemporary data-driven research, informed consent and information continues to stand as a fundamental ethical imperative in clinical research. In addition to serving as consent for participation in a clinical trial, informed consent also represents a mechanism through which an individual can express their preferences and authorize the use of their data (28).

In the context of the FACILITATE project, it is essential to clarify that the obligation to inform data subjects of their rights under Article 13(2)(b) of the GDPR is universal, irrespective of the legal basis applied. This is a requirement, but in practice, after data is collected, there are no practical means to contact participants in clinical trials. This includes rights such as access (Article 15) and data portability (Article 20).

Our emphasis on informed consent within clinical trials aims to highlight the necessity of explicitly communicating these rights to participants, given the sensitivity of clinical trial data and the significant implications of data handling practices.

Data processing under the clinical trial regulation does not require consent, as the regulation itself serves as legal basis for processing. However, requirements for data processing under the clinical trial regulations are only defined for the aims of the clinical trial itself (data collected for the purpose of the trial, safety, etc.) therefore, it is unclear how to handle different aspects such as the return of results to patients after the clinical trial ends and the secondary use of data collected during the clinical trial (not covered by the clinical trial regulation). The clinical trial regulations ask for consent for those activities, though this is not consent under the GDPR as cleared by EDPB.

Therefore, in FACILITATE, we do not consider consent as the preferred GDPR legal basis (this may be country specific) but rather as an ethical requirement to enable full autonomous choices with regard to both secondary use and return. The form of ongoing information and consent we envisage, though, will also be compliant with GDPR for the countries that require it as a legal basis (for example, Italy). This approach ensures that consent processes are compliant with regulatory mandates and better inform participants about the extent of their rights fostering greater transparency and trust.

It is clearly critical to articulate the distinction between the consent required for clinical trial participation under the Clinical Trials Regulation and the consent for further processing and return (that may or may not be a legal basis within the GDPR depending on the different legal frameworks).

The ethical framework developed by FACILITATE advocates for a nuanced understanding that, while the consent for trial participation primarily addresses data use within the trial, consent for return of data should cover broader ethical considerations and data protection principles that extend to subsequent uses.

This also means that within the project the importance of other legal bases, such as public interest or the legitimate interests of the controller, is explored and especially how these bases influence the practicalities of returning data to patients and the broader implications for patient autonomy, trust for secondary uses, and trustworthy environments in general. Our envisioned framework is vital for aligning the consent processes with both ethical considerations and legal standards, whereby this dual approach ensures both compliance and respects the autonomy and individual preferences of participants, thus reinforcing the ethical framework of the FACILITATE project.

A participant-centric approach cannot be realized without considering the individual’s voice, making informed consent an indispensable component. Therefore, while retaining the informed consent process, it should be reimagined to accommodate personalized decisions and possess the flexibility to adapt over time. FACILITATE’s processes and ethical frameworks should thus facilitate the return of clinical trial data while accounting for evolving individual preferences.

Transparency regarding data use is paramount, and interactive consent models utilizing information technology (IT) have been proposed to facilitate continuous communication and information dissemination, allowing participants to modify their consent choices and stay informed about data usage (22). To achieve its objectives, FACILITATE is building a participant-centric prototype process known as “FACILITATE Consent,” which aligns with participant expectations expressed thus far in the project. This consent should provide updated information, allow participants to modify preferences, and incorporate oversight mechanisms.

By aligning the informed consent process with GDPR’s stipulations on data subject rights (29), FACILITATE ensures that participants are not just passive subjects in a trial but active participants in their data management. This approach not only enhances participant trust and trial integrity but also aligns with broader ethical principles of respect, autonomy, and transparency (30–32).

## Actionable recommendations

To support this process, FACILITATE has developed a draft ethical framework that outlines the ethical principles and procedures designed to facilitate a participant-centric approach in returning individual clinical trial data. Its purpose is to guide industry, academia, and all stakeholders involved in clinical trials in negotiating the intricate terrain of returning clinical trial data to patients. The framework is applicable to the process of returning clinical trial data to participants during and after a clinical trial under the EU Clinical Trials Regulation. The primary objective of this framework is to ensure the ethical management of returning individual clinical trial data to study participants both during and after the clinical trial. Specifically, the framework aims to:

- Define the key principles that should govern the individual return of clinical trial data to patients.
- Identify and address potential risks to participants and their families associated with the return of clinical trial data.
- Establish a clear and accountable patient-centric process for the ethical return of results throughout and following the clinical trial.

Within the draft ethical framework, the ethical return of clinical trial data to study participants is shaped by the Substantive Principles described below. In the process of returning clinical trial data to participants, the Procedural Principles must also be adhered to. It is important to note that no single principle takes precedence over another; instead, a harmonious balance among these principles is essential. The accompanying draft framework delineates the practical application of these principles and how balance among them is maintained (Tables 2, 3).

**Table 2:** Substantive principles.

Rights and respect for individuals and wider society	Individuals have the right to make autonomous and informed decisions. This includes what, if any, clinical trial data should be returned to them. The return of clinical trial data must respect the right of study participants to be informed, their right to access or not their data, and respect a participant’s preferences on the return of clinical trial data. The return of data should not be contingent on the participant’s completion of the clinical trial.
Beneficence	The return of clinical trial data must be guided by a consideration of the best interests of the study participant.
Non-maleficence	Clinical trial data shall be returned to participants in a manner that maximizes any benefits and minimizes any risks to participants.
Privacy and confidentiality	The return of clinical trial data must respect the individual subject’s privacy and the confidentiality of their data. Any limitation of that right must be necessary, limited, proportionate, accountable, and transparent with protections in place to continue to safeguard the subject’s privacy and confidentiality.
Autonomy	Autonomy is a fundamental ethical principle in clinical trials that emphasizes the right of individuals to make informed decisions about their participation.
Utility	The return of clinical trial data must be of value to the study participant (this should be subjective rather than objective, e.g., actionable).
Empowerment	Study participants should be empowered to make informed decisions about their healthcare. The individual clinical trial data returned and the process for returning it, including who returns the clinical trial data, should enable this empowerment.
Public value	The primary goal of clinical research is the production of generalizable knowledge for the patients who will benefit from the scientific knowledge. Clinical trials are critically important in improving the public’s health. Any return of clinical data, and the timing of that return, must be balanced against the scientific integrity of the clinical trial.
Data custodianship	To return high quality and reliable data to a participant, it is essential to have control over the process that generates the results themselves. Traceability of the processes that generated the results can ensure the accuracy and pertinence of the data that is returned to the right clinical trial participant.
Justice	Returning clinical trial data must be done in a manner that is lawful, fair and just.

**Table 3:** Procedural values.

Transparency	The process to be followed in the return of clinical trial data must be clear and explained to the study participants at the time of the informed consent. It must be clear to study participants the type of data that will be returned and when. The process to be followed if a participant changes their preferences must be clear and communicated to the participant.
Accountability	It must be clear who is responsible for ensuring that clinical trial data is returned to participants.

The FACILITATE framework incorporates a blend of traditional bioethical principles, such as beneficence, non-maleficence, and justice, alongside newer concepts like empowerment and utility. This integration reflects a broader move in clinical research ethics to accommodate evolving perspectives on participant interaction and data management. The ethical principle of empowerment closely relates to the classic principle of respect for autonomy. Both empowerment and respect for autonomy prioritize giving individuals control over their own decisions. While autonomy emphasizes an individual’s right to make choices free from coercion and with sufficient information, empowerment goes a step further by actively enhancing one’s capacity to make those choices. This can include providing tools, resources, education, and support to ensure individuals are not only making decisions independently but are also equipped and capable of doing so effectively. By integrating the principle of empowerment with respect for autonomy, ethical practices and policies should not only protect individual choice but also actively enhance individuals’ ability to participate fully and effectively in decisions that affect their lives. This combination is particularly vital in settings like healthcare, research, and social services, where decisions have significant personal and communal impacts (33–35).

The principle of “utility” in the context of returning clinical trial data asserts that the data returned must provide subjective, actionable value to the participant. This principle raises significant questions regarding the assessment of value and its potential conflicts with the data protection legal framework, particularly under the General Data Protection Regulation (GDPR). Under Article 15 of the GDPR, individuals can access their personal data from data controllers without needing to justify their request. This provision enhances transparency and empowers individuals to control their information. The principle of utility, which advocates for returning data considered subjectively valuable, complements rather than conflicts with this right. While the right of access is unconditional, the utility principle serves to proactively return data in situations where participants may not understand what to request or the potential significance of the data to their health (36).

Also, the principle of utility aims to enhance the relevance and impact of returned data for participants, promoting a more participant-centered approach in clinical trials. However, it must be implemented with a clear understanding and adherence to GDPR requirements, ensuring that participants’ legal rights to access their data are uncompromised. By maintaining this balance, clinical trials can achieve both ethical integrity and compliance with data protection laws, ultimately fostering a more trustworthy and effective research environment (30–39).

In addition to establishing more traditional and newer ethical principles, the FACILITATE ethical framework sets out a guide to implementing these principles in practice. The principles and guidance on implementation seek to ensure that participants data is returned while being flexible and adaptable to the differing contexts in which clinical trials occur. At this stage of the developing implementing framework, FACILITATE is focusing on these critical elements.

First, the implementing framework requires the establishment of transparent and accountable processes. To achieve this, it provides that the roles and responsibilities of key individuals in the return of clinical data decision-making process shall be identified, and there shall be clear, transparent and ongoing information to participants throughout the entire process on the return of data.

Second, participant information and the decision process are critical. The implementing framework requires that during the clinical trial informed consent process, participants shall be informed that the purpose of the clinical trial is to identify generalizable results based on statistical inference and not individual care. This must be planned in the protocol whether they may or may not receive individual data during the trial, depending on the type and set-up of the trial (blinded or not etc.) Participants shall also be clearly informed that during and after the clinical trial, data may emerge that may be relevant for their health and the modalities foreseen for potential recontact in those cases. Participants shall be clearly informed on their rights with regard to access to their individual data of all medical tests if they consent to it. Participants shall as well be informed that data may arise that can impact decisions on their healthcare during the clinical trial. Data that can lead to decisions that are lifesaving, urgent, or actionable must already be returned to the participant during the clinical trial in accordance with the clinical trial regulation. This applies even if the return of individual data can result in the unintentional unblinding of the individual and risks the integrity of the overall trial. Participants shall be informed that data not urgent but actionable may arise. They shall be informed about these data, unless for reasons that may include preserving the integrity of the clinical trial. All data shall be returned to participants after the clinical trial. Sensitive data shall be returned in the appropriate manner. Study participants shall be informed who is responsible and how the data will be returned to them (e.g., in the form of a letter, through a portal, by their health care practitioner, study team member, etc.). Study participants shall be informed that they shall receive the general study results at the end of the clinical trial. This can be in several different methods that can include an invited meeting, an online seminar, or information printed on a website. What is important is that all patients are made aware of where and how the general study results will be returned, that the information is clear and understandable, and that patients have the opportunity to ask questions.

Third, at this stage of the developing FACILITATE processes, it is envisaged that a participant information and decision tool will the developed to both return individual clinical trial data and possibly enable the management of the secondary use of clinical trial data. The ethical framework currently provides that individual data after the clinical trial shall be communicated to participants through a participant tool through which the participant and their physician(s) can access their data. Participants shall be informed that it is their responsibility to ensure that their contact details are kept up to date on this tool. They shall be informed that failure to do so can impact their ability to receive ongoing information.

This ethical framework is currently in draft form and will be updated throughout the life cycle of the project as processes development. Critical throughout this will be more robust ongoing patient engagement to ensure that the processes are in line with patient expectations. This is achieved by patients and their advocates providing ongoing insights. Regular feedback sessions, both online and in-person, are held to maintain an open line of communication with participants. Additionally, surveys and questionnaires are be distributed periodically to gather a broader perspective on the participants’ views regarding data handling and ethical practices. Integrating patient representatives directly into the work packages further ensures that participant perspectives are continuously considered in decision-making processes. Moreover, the use of innovative digital engagement tools will allow participants to interactively view, comment on, and vote on proposed changes, providing a real-time feedback loop. This comprehensive strategy aims to enhance trust, and alignment with participant needs and expectations throughout the lifecycle of the project.

## Conclusion

Clinical trial data carries significant untapped potential, often left underutilized beyond the trial itself, with limited data returned to patients. Enabling this data use for future research and returning the clinical trial data to participants calls for a paradigm shift in our approach before, during, and after clinical trials. This shift should address legal and ethical barriers hindering data return of individual clinical trial data throughout and after the trial. To make meaningful progress in this domain, we must depart from conventional practices and thinking that often obstruct effective data return and reuse, going beyond what it strictly requires, looking forward and building a trusted environment where patients can feel safe and protected with a say about their health data and control over it. The ethical framework outlined in this draft represents the strategy that FACILITATE is employing and striving towards to achieve a substantive ethical, legal, and meaningful data return.

## Author contributions

CS: Writing – original draft, Writing – review & editing. JB: Funding acquisition, Writing – original draft, Writing – review & editing. DM: Writing – original draft, Writing – review & editing.

## Footnotes

^1^ www.transceleratebiopharmainc.com

^2^ mrctcenter.org
